# Artificial intelligence in fusion protein three‐dimensional structure prediction: Review and perspective

**DOI:** 10.1002/ctm2.1789

**Published:** 2024-08-01

**Authors:** Himansu Kumar, Pora Kim

**Affiliations:** ^1^ Department of Bioinformatics and Systems Medicine McWilliams School of Biomedical Informatics The University of Texas Health Science Center at Houston Houston Texas USA

**Keywords:** AI, AlphaFold2, deep learning, fusion protein structure, protein structure prediction, RoseTTAFold

## Abstract

Recent advancements in artificial intelligence (AI) have accelerated the prediction of unknown protein structures. However, accurately predicting the three‐dimensional (3D) structures of fusion proteins remains a difficult task because the current AI‐based protein structure predictions are focused on the WT proteins rather than on the newly fused proteins in nature. Following the central dogma of biology, fusion proteins are translated from fusion transcripts, which are made by transcribing the fusion genes between two different loci through the chromosomal rearrangements in cancer. Accurately predicting the 3D structures of fusion proteins is important for understanding the functional roles and mechanisms of action of new chimeric proteins. However, predicting their 3D structure using a template‐based model is challenging because known template structures are often unavailable in databases. Deep learning (DL) models that utilize multi‐level protein information have revolutionized the prediction of protein 3D structures. In this review paper, we highlighted the latest advancements and ongoing challenges in predicting the 3D structure of fusion proteins using DL models. We aim to explore both the advantages and challenges of employing AlphaFold2, RoseTTAFold, tr‐Rosetta and D‐I‐TASSER for modelling the 3D structures.

**Highlights:**

- This review provides the overall pipeline and landscape of the prediction of the 3D structure of fusion protein.
- This review provides the factors that should be considered in predicting the 3D structures of fusion proteins using AI approaches in each step.
- This review highlights the latest advancements and ongoing challenges in predicting the 3D structure of fusion proteins using deep learning models.
- This review explores the advantages and challenges of employing AlphaFold2, RoseTTAFold, tr-Rosetta, and D-I-TASSER to model 3D structures.

## Graphical abstract

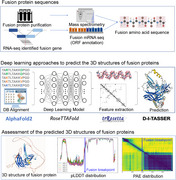

## 1 INTRODUCTION

A fusion protein is composed of the combination of at least two partial protein domains, each encoded by separate genes, merging to undergo joint transcription and translation. Precisely predicting a protein's three‐dimensional (3D) structure is essential for the accuracy of subsequent drug discovery processes. This includes predicting protein functions, studying protein–protein interactions, finding inhibitors, designing antibodies and analyzing protein–ligand interactions. ^1^, ^2^ Prediction of the 3D structures of proteins has mostly relied on protein sequence data and their structural homology.^3^ There are huge efforts to understand better and study the 3D structures of wild‐type (WT) proteins. However, we lack the effort to predict the fusion protein 3D structures and to have enough knowledge. Fusion proteins are created when fusion transcripts, resulting from the transcription of fusion genes between two different genes in DNA, are translated. This process is triggered by chromosomal rearrangements due to DNA double‐strand breaks. These fusion proteins, which are the combined globular form of two different protein structures, including the major functional domains or partial, can result in novel proteins with new functions or regulations in the cells, thereby causing disease.^4^ Many fusion proteins have been utilized as therapeutic targets in cancer treatments.^5^, ^6^, ^7^, ^8^

Fusion genes can impact several functional mechanisms, including cell signal transduction and the activation of downstream target genes by transcription factor (TF) fusion proteins. They can also lead to the loss of protein–protein interactions and the upregulation of oncogenic fusion proteins due to the loss of miRNA regulation. Additionally, TF fusion proteins can bind to promoters, increasing the expression of oncogenic downstream effectors. Fusion proteins can also result in the loss of subunits within cellular regulatory complexes.^9^ Among these, kinase fusion proteins were the most studied and targeted fusion proteins. However, in reality, even for these most studied kinase fusion proteins, there is a shortage of complete 3D structures for fusion proteins. Multiple reasons can explain this lack of the 3D structures of fusion proteins. Before the artificial intelligence (AI)‐based prediction model, protein modelling methods were based on force‐field optimization and multiple energy functions.^10^ These optimizations describe atomic interactions in proteins as the combined effect of various bonds, including electrostatic interactions, non‐covalent van der Waals forces, hydrogen bonds and covalent bonds between atoms.^11^ So far, it has been challenging to find studies that predict the complete 3D structures of fusion proteins, as opposed to the structures of WT proteins. We previously faced a challenge due to the absence of protein sequences for fusion proteins, having only the genomic fusion breakpoints. To address this, our group recently investigated the open reading frames (ORFs) of the full‐length fusion transcript sequences from ∼121 000 human fusion genes. This information has been made available through FusionGDB2.0.^12^, ^13^ Furthermore, the most targetable driver fusion proteins (i.e. kinase fusion proteins) have very long protein sequence lengths, so the crystallization of whole fusion protein structure might be difficult.^14^ In silico protein structure design often relies on existing structural templates, but there are no structural templates for the fusion proteins. Therefore, predicting accurate 3D structures of fusion proteins remains a challenge using typical protein structure prediction methods.

For example, for the in silico protein structure design of a fusion protein, we need to rely on existing structural templates of one of the protein partners in the WT form. For the fusion protein BCR‐ABL, the ABL1 kinase domain acts as a well‐established template, with its structure available in the Protein Data Bank (PDB) such as 2HYY, 1OPJ, 1OPL, 1OPK, 2F4J, 2GQG, 2HZF, 2I4I, 2OIQ and 2OIR.^15^, ^16^ Similarly, for the TF fusion protein EWSR1‐FLI1, the FLI1 component's structure is known and is available in the PDB with entries like 1FLI, 4JYZ, 4JZD, 4JZF, 4JZG, 5L9X and 4JZH. These known structures provide a foundational framework for modelling and predicting the architecture of the respective fusion proteins. In this scenario, to predict the 3D structures of fusion proteins BCR‐ABL1 and EWSR1‐FLI1, researchers have to rely on ABL1 and FLI1 structure templates, respectively. Overall, the prediction of whole fusion protein structures is a challenging problem because of size and complexity/ambiguities, lack of experimental data, conformational flexibility and lack of suitable computational methods. Recently, protein structure prediction tools using AI technologies, like AlphaFold and RoseTTAFold, have attracted the attention of researchers to predict 3D structures as compared to classical force‐field–based models.^17^, ^18^

The 3D structure of fusion proteins is crucial in the computer‐aided drug design process. Accurate 3D models of fusion oncoproteins are vital for understanding the binding interactions between these proteins and potential drug molecules, a key step in developing new therapeutic agents. For example, the BCR‐ABL1 fusion protein in leukemia has been targeted by multiple kinase inhibitory small molecules, such as imatinib, bosutinib, nilotinib, dasatinib and ponatinib. These drugs have been approved by the FDA for treating various adult cancers, including leukemia, lymphoma, chronic myeloid leukemia, thyroid cancer, pancreatic cancer, breast cancer, lung cancer, ovarian cancer, gastrointestinal stromal tumours, renal cell carcinoma, hepatocellular carcinoma and prostate cancer.^19^, ^20^, ^21^, ^22^, ^23^, ^24^, ^25^ Tumorigenic functions of multiple kinase fusion genes, including *ABL, ALK, ROS1, RET and NTRK*, are extensively targeted for cancer therapeutics.^26^, ^27^ However, many fusion proteins are still awaiting therapeutic targeting through various mechanisms. For example, EWSR1‐FLI1 is a fusion protein between EWSR1 RNA‐binding protein and FLI1 TF, leading to the onset of Ewing sarcoma.^28^ Overall, 100% of Ewing sarcoma patients have EWSR1 fusion proteins.^28^ FLI1 has been targeted as the receptor of Ewing sarcoma for small inhibitory molecules for its DNA‐binding domain. However, there are currently no approved drugs specifically and effectively targeting the aberrant function of EWSR1‐FLI1 fusion oncoprotein in Ewing sarcoma patients.^20^, ^29^, ^30^ From these reasons, currently, we urgently need the knowledge of the whole 3D structures so that we can get the accurate structures of fusion proteins and initiate the drug designing process.

**FIGURE 1:**
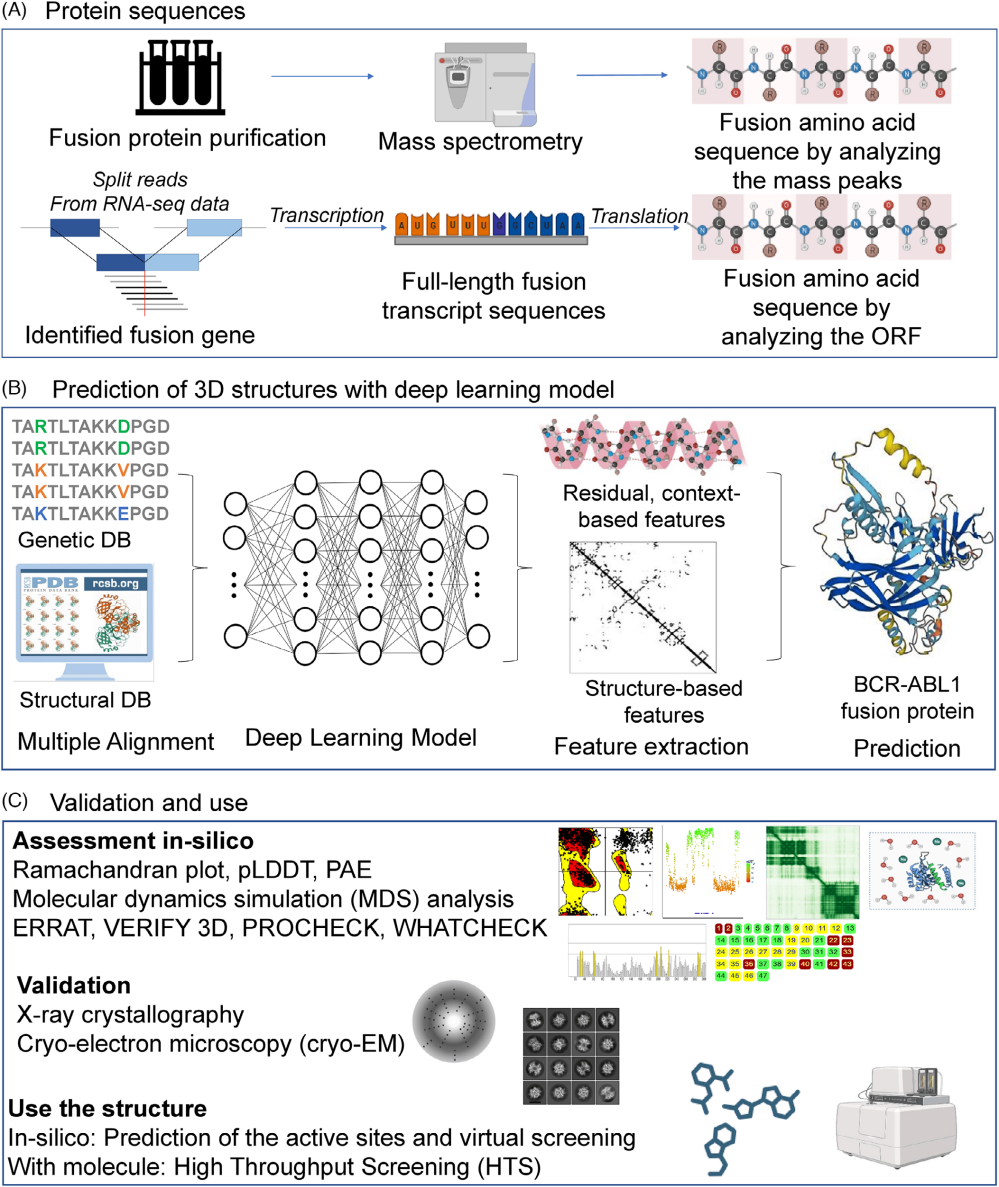
Overview of deep learning model to predict the fusion protein structure prediction. (A) Protein sequences. (B) Prediction of three‐dimensional (3D) structures with deep learning model. (C) Assessment in silico, Validation and use of the predicted 3D structure.

Recent advances in AI, particularly with tools like AlphaFold and RoseTTAFold, have significantly improved the accuracy of protein 3D structure prediction. This progress is particularly beneficial for predicting the structures of fusion proteins.^31^, ^32^ Applying these AI tools to the fusion protein sequence information from FusionGDB 2.0, recently, we developed a novel computational pipeline and established a resource for human fusion proteins named FusionPDB.^9^, ^33^, ^34^ FusionPDB provides ∼42K fusion protein sequences, 3D structures of ∼3500 fusion proteins using AlphaFold2 and evidence of reliable 3D structures. In this review, as shown in Figure 1, we share our understanding and challenges of the fusion protein structure prediction problem based on our experience using AI approaches such as AlphaFold and RoseTTAFold.^31^, ^32^ Figure 1 offers a systematic overview of the workflow for predicting and validating fusion protein structures using deep learning (DL) models. Panel A outlines the process from identifying the fusion gene using RNA‐seq data to determining the fusion protein sequence through mass spectrometry and ORF analysis. Panel B illustrates the prediction of 3D structures, starting with data from genetic databases and multiple sequence alignments (MSAs), feeding into a DL model that integrates structural and residual context‐based features to predict the fusion protein structure, exemplified by the BCR‐ABL1 protein. Panel C details the validation of the predicted structures using in silico assessment tools such as Ramachandran plots, pLDDT and PAE, supplemented by molecular dynamics (MD) simulations and structural validation software like ERRAT and PROCHECK. Further validation is carried out using experimental methods such as X‐ray crystallography and cryo‐electron microscopy (cryo‐EM). The refined structure is then used in active site prediction and virtual screening to get the molecular interaction information. We hope that our review and identification of challenges in fusion protein structure prediction will aid in advancing the development of fusion protein‐targeted therapeutics and in improving the design of synthetic proteins on demand.

### 1.1 Fusion proteins used in this review for the in silico prediction of 3D structures

To explore and discuss the AI‐based prediction of fusion protein structures with real fusion gene examples, we have considered the top four most cited fusion proteins (BCR‐ABL, EML4‐ALK, TMPRSS2‐ERG and PML‐RARA). Table S1 catalogues the in‐frame fusion genes identified in the study, listing the 5′ and 3′ gene partners and the corresponding number of articles reported in PubMed. This tabular representation helps in understanding the prevalence and research coverage of specific gene fusions. The BCR‐ABL fusion protein is a chimeric protein resulting from a genetic abnormality commonly found in acute lymphoblastic leukemia and chronic myeloid leukemia.^27^ This fusion protein is essential for the development and progression of leukemia. The *ABL1* gene encodes a tyrosine kinase enzyme that regulates cell growth and division. However, when combined with the *BCR* gene sequence, the resulting BCR‐ABL fusion protein has constitutive kinase activity that is unregulated, and promoting uncontrolled cell proliferation.^35^ To identify relevant PDB entries for the BCR‐ABL fusion protein, I utilized the Advanced Search feature on the PDB website. The search was configured to filter entries based on specific criteria under the ‘Structure Attributes’ section. I used two exact phrase queries: ‘BCR’ and ‘ABL1’ within the ‘Citation Title’ field. This ensured that only those PDB entries where the citation title contains both ‘BCR’ and ‘ABL1’ were retrieved. This approach was chosen to precisely locate structures related to the BCR‐ABL fusion protein, ensuring a focused and accurate selection of relevant data for this study. The PDB database contains eight entries for the human fusion protein BCR‐ABL1; however, it is important to note that none of these entries represent the complete structure of the fusion protein. Instead, all of them correspond to specific domains of either the BCR or ABL partner proteins. The EML4‐ALK fusion protein is another chimeric protein that occurs in certain types of cancer, most notably non‐small cell lung cancer (NSCLC).^36^, ^37^ The EML4 gene typically encodes a protein associated with microtubule structures within cells, whereas the ALK gene encodes a receptor tyrosine kinase that plays a role in cell growth and differentiation. The abnormal activity of the EML4‐ALK fusion protein is crucial in the development and progression of a subset of NSCLCs. This fusion protein drives uncontrolled cell growth, proliferation, and contributing to the formation of tumours. In the PDB database, similarly, for the EML4‐ALK fusion protein, I used the Advanced Search feature on the PDB website with the ‘Citation Title’ field set to contain the exact phrases ‘EML4’ and ‘ALK’. This search method yielded three relevant PDB entries for the EML4‐ALK fusion protein, which were subsequently analyzed in this study, and they exclusively represent specific domains of either the EML4 or ALK partner proteins.

Similarly, the TMPRSS2‐ERG fusion protein results from a genetic rearrangement that occurs in certain types of cancer, particularly prostate cancer.^38^ The TMPRSS2 gene typically encodes a serine protease enzyme that is involved in various cellular processes.^39^ The abnormal expression of the TMPRSS‐ERG fusion protein has been implicated in the development of a subset of prostate cancers. Likewise, I searched for the TMPRSS2‐ERG fusion protein by setting the ‘Citation Title’ field to contain the exact phrases ‘TMPRSS2’ and ‘ERG’. This search did not yield any relevant PDB entries for the TMPRSS2‐ERG fusion protein. Furthermore, the PML‐RARA fusion protein is formed as a result of a specific chromosomal translocation that occurs in acute promyelocytic leukemia, a subtype of acute myeloid leukemia.^40^ The PML gene encodes a protein involved in various cellular processes, including the regulation of cell growth and apoptosis. The RARA gene encodes a retinoic acid receptor, which is a TF that regulates gene expression in response to retinoic acid. Using the Advanced Search feature on the PDB website, I searched for the PML‐RARA fusion protein by setting the ‘Citation Title’ field to contain the exact phrases ‘PML’ and ‘RARA’. This search resulted in two relevant PDB entries for the PML‐RARA fusion protein.

In this study, we employed a range of AI‐based and traditional protein structure prediction models for predicting the 3D structures of fusion proteins. Table 1 provides a summary of the key features and references for each prediction tool. These tools, including AlphaFold2, RoseTTAFold and trRosetta, utilize advanced DL techniques to predict protein structures with high accuracy. Traditional methods, like MODELLER and I‐TASSER, rely on homology modelling and iterative threading assembly refinement, respectively, to build 3D models from amino acid sequences. The combination of these tools provides a comprehensive approach to accurately model the structures of fusion proteins, thereby aiding in understanding their functional and structural properties.

**TABLE 1:** Features and references for artificial intelligence (AI)‐based and other protein structure prediction models providing an overview of various tools used for predicting protein structures, detailing their features and specific references.

Prediction tool	Features	Reference
**AI‐based protein structure prediction model**		
AlphaFold2	AI‐based tool that predicts 3D protein structures from amino acid sequences with high accuracy, leveraging deep learning and MSAs It uses deep learning with attention mechanisms and a transformer architecture to predict 3D structure	^31^
RoseTTAFold	It is a deep learning–based prediction tool that utilizes a three‐track neural network architecture to simultaneously consider sequence, distance and coordinate information. It integrates evolutionary information and physical constraints to accurately predict protein 3D structures from sequences	^18^
trRosetta	It uses deep learning to predict inter‐residue orientations and distances from MSA. It then assembles these predicted geometric constraints into 3D structures using a fast, gradient‐based energy minimization protocol	^41^
D‐I‐TASSER	A deep learning–based extension of I‐TASSER that integrates deep neural networks for predicting inter‐residue distance and contact maps. These predictions are then used to guide the iterative threading assembly refinement process to model protein 3D structures	^42^
**Other protein structure prediction model**		
MODELLER	A tool for homology or comparative modelling of protein three‐dimensional structures. It generates models by satisfying spatial restraints derived from the alignment of target sequences with known protein structures	^43^
I‐TASSER	Prediction on the sequence‐to‐structure‐to‐function paradigm. It constructs 3D models by iteratively threading protein sequences through a representative PDB structure library, followed by atomic‐level structure refinement	^44^
Phyre2	A suite for protein modelling, prediction and analysis. It uses advanced homology modelling techniques, combining multiple sequence alignment and threading methods, to generate 3D models based on known protein structures	^45^
ROSETTA	Ab initio modelling and has template‐based modelling capabilities. It uses fragment assembly and energy minimization techniques to predict protein structures, design new proteins and model protein–protein interactions	^46^
SWISS‐MODEL	SWISS‐MODEL is a web‐based automated protein structure homology‐modelling tool that predicts 3D structures based on experimentally determined structures of related proteins. It utilizes template‐based modelling to build accurate structural models, aiding in understanding protein function and guiding experimental design	^47^
RaptorX	It utilizes deep learning techniques to accurately predict protein 3D structures, contact maps and solvent accessibility from amino acid sequences	^48^
HHpred	It uses hidden Markov models (HMMs) to detect remote homologs and infer structural and functional information based on sequence alignments	^49^
CNFpred	CNFpred is a computational tool designed to predict the native conformational state of proteins using a combination of sequence information and advanced neural network algorithms to model complex protein folding mechanisms	^50^
CEthreader	It uses a combination of profile–profile alignment and energy‐based scoring functions to predict the 3D structure of a protein by aligning its sequence with known structures in a database	^50^
ResPre	ResPre is a protein structure prediction tool that employs deep residual neural networks to predict inter‐residue distance and orientation for accurate modelling of protein tertiary structures	^51^
ProALIGN	Directly learning alignments for protein structure prediction via exploiting context‐specific alignment motifs. ProALIGN is a protein structure alignment tool designed to compare and align 3D structures of proteins, using advanced algorithms to identify structural similarities and differences	^52^

Abbreviations: MSA, multiple sequence alignment; PDB, Protein Data Bank.

## 2 THE STARTING MATERIALS: FUSION PROTEIN SEQUENCES

To identify fusion protein sequences, two methods are illustrated in Figure 1A. The first approach involves obtaining the fusion protein sequences from the purified fusion protein sample. After purification of the fusion proteins from the cells, we can use the mass spectrometry to identify the fusion peptide sequence by analyzing the mass spectrometry signals. However, to have the purified fusion proteins is not easy. Designing an effective purification strategy that maintains the biological activity of the fusion protein is challenging. The strategy must account for the properties of both the tag (if used) and the protein of interest, including their isoelectric points, hydrophobicity and affinity for different ligands. Second approach is the prediction from the genomic breakpoint information by analyzing the unmapped split reads between two genes from the RNA sequencing data. Then, for the identified genomic breakpoints, we first check the exon junction match because the most of the genomic breakpoints are located in the intron regions than exon regions. For the exon junction aligned cases, we check their ORFs and only remain the in‐frame fusion genes. For in‐frame fusion genes, we create the full‐length transcript sequence by considering multiple gene isoforms. We then select the longest amino acid sequences, from all potential six frame–based methods by ORFfinder, as the fusion protein sequence.^53^

## 3 COMPUTATIONAL PREDICTION OF THE 3D STRUCTURES OF FUSION PROTEINS

There are multiple challenges for predicting the 3D structure of fusion protein. Fusion protein has complex architecture with multiple domains and linkers that can interact with each other in various ways.^54^, ^55^ Therefore, it is difficult to accurately predict the folding of the fusion protein. Furthermore, there is lack of experimental data for fusion proteins in available database, which makes it challenging to validate computational predictions. Next, fusion proteins have novel combinations, by combining domains and sequences from different proteins that were not previously studied, making it difficult to predict the folding and stability of the protein. Most of all, conformational changes in fusion proteins can undergo conformational changes in response to changes in the environment or binding to other molecules. These changes can be difficult to predict computationally. Last, predicting the 3D structure of a fusion protein can be computationally demanding, requiring advanced algorithms such as DL and significant computing resources as shown in Figure 1B. However, various groups had used computational tools and approaches to predict the 3D structure of fusion proteins, which include homology modelling, MD simulations and machine learning algorithms.^56^, ^57^ Experimental techniques, such as nuclear magnetic resonance (NMR) spectroscopy and X‐ray crystallography, can be used to get structural information of fusion proteins, which can be used to validate computational predictions (Figure 1C).^58^

### 3.1 Current scenario of computational tools of structure prediction

**FIGURE 2:**
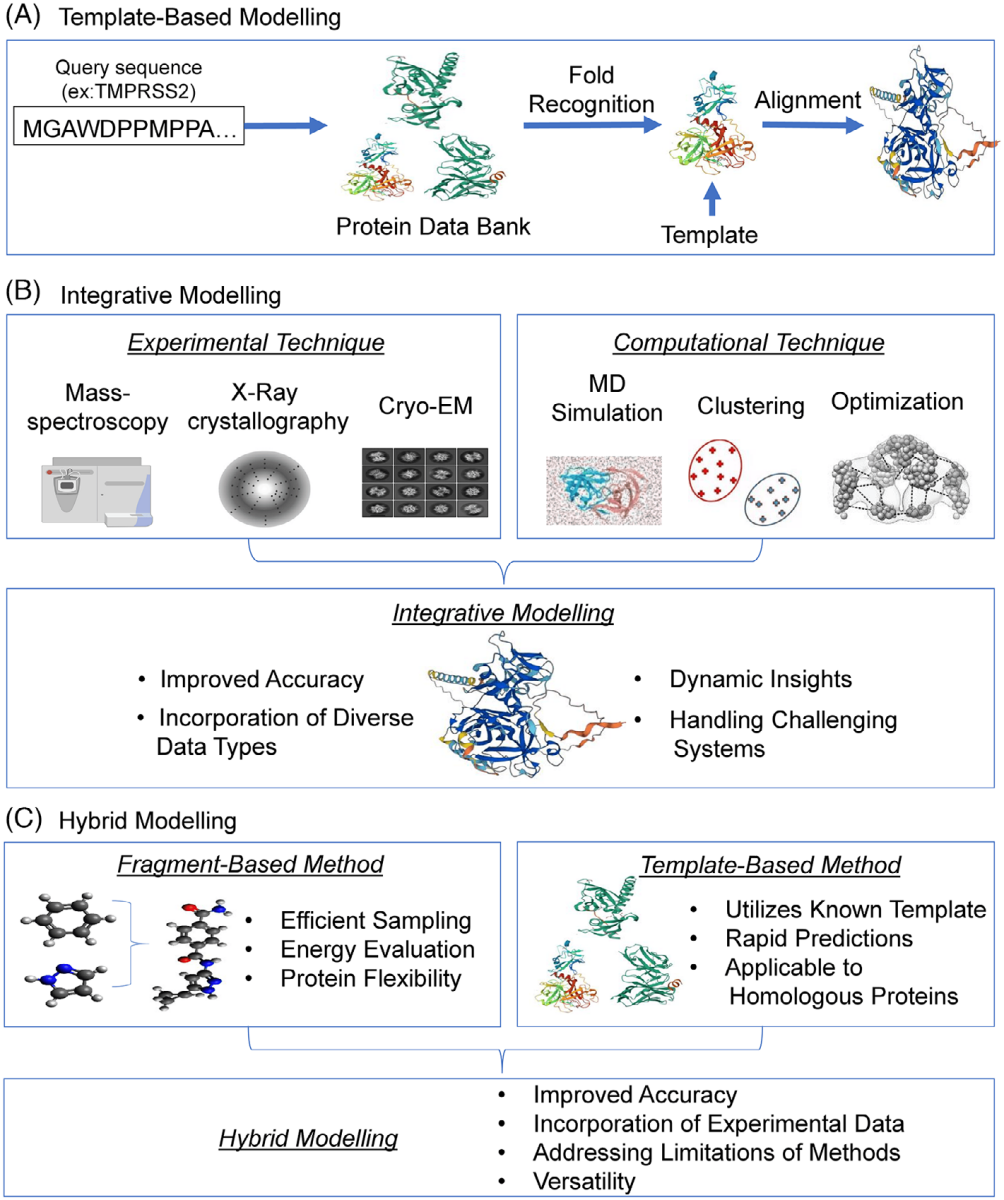
Traditional protein structure prediction modelling: (A) Template‐based, (B) integrative, (C) Hybrid.

Before the advancement of the AI approaches, in silico structural biology works, such as 3D structure prediction, MD simulations and protein–protein interaction predictions, were performed on the basis of the homology modelling, force‐field optimization and energy function.^59^, ^60^, ^61^, ^62^ Some of the well‐known modelling methods, such as fragment‐based modelling (FBM), template‐based modelling (TBM), integrative method and hybrid method, have been discussed below and shown in Figure 2.

### 3.2 Fragment‐based modelling

Fragment assembly methods in protein structure prediction utilize local sequence–structure correlations to construct tertiary models by combining sequence fragments. These methods face limitations in exploring diverse conformations due to fragment libraries insufficient representation of native‐like features, especially in loop regions. Fragment‐based methods break a protein sequence into smaller segments, or fragments, and use these fragments to predict the overall structure.^63^ This approach has been successful in predicting the structures of larger proteins and protein complexes.^64^ Similarly, it has been applied to predict the structures of fusion proteins, which are proteins composed of two or more domains or subunits from different proteins.^56^, ^65^, ^66^ Fragment assembly methods leverage known sequence–structure relationships to assemble the tertiary structure of proteins from smaller fragments. For fusion proteins, fragment assembly can effectively predict how these segments might fold and interact based on their local sequence context.

### 3.3 Template‐based modelling (TBM)

It relies on the availability of homologous protein structures as templates to model the structure of a target protein.^67^, ^68^ TBM is a method for predicting the 3D structure of proteins by using known protein structures as templates. It aligns a target protein sequence to these templates and generates a model based on this alignment as shown in Figure 2A. TBM is particularly effective when a closely related structure is available, making it a preferred approach for proteins with homologous structures already determined. Recent advancements in TBM include the use of DL methods to improve template selection and refinement.^69^ HHpred,^49^ DeepThreader,^70^ CEthreader and NDThreader^70^, ^71^ represent a class of widely‐used AI‐based tools in the domain of TBM for protein structure prediction. In the case of fusion protein, when parts of a fusion protein resemble known structures, TBM can model these segments by aligning them to existing templates. For unique conformations specific to fusion proteins, advancements in AI and DL are still needed within TBM, to enhance the selection and refinement of templates, and improve the prediction accuracy even when direct homologs are sparse. Fusion proteins may combine domains or full‐length proteins from different sources, for which no single template exists that encompasses the entire fusion construct.

### 3.4 Integrative methods

Integrative methods combine multiple sources of experimental and computational data to predict protein structures. These methods include X‐ray crystallography, cryo‐EM, NMR spectroscopy and bioinformatics techniques. By combining these different data sources, integrative methods can produce more accurate protein structure predictions. This approach integrates experimental data from small‐angle X‐ray scattering measurements with computational techniques such as ab initio modelling, MD simulations and TBM to produce a high‐resolution model of the fusion protein, as illustrated in Figure 2B. The integrative approach allowed researchers to take advantage of the strengths of each method and improve the accuracy and reliability of the final structure prediction. This approach is particularly valuable for fusion proteins, which may not be fully captured by single‐method analyses.

### 3.5 Hybrid methods

Hybrid methods combine multiple computational approaches to predict protein structures. For example, a hybrid method might use a fragment‐based approach to predict local protein structure and then use a template‐based approach to refine the overall structure. These techniques initially use homology modelling to create a preliminary structural model of the fusion protein based on the known structures of its individual domains. They then used de novo folding to refine the model and improve its accuracy and reliability. A well‐known example of a hybrid protein structure prediction method is Rosetta. Rosetta is an extensive suite of algorithms designed for computational modelling and analysis of protein structures. It uniquely combines ab initio modelling, TBM, and machine learning techniques as shown in Figure 2C.

## 4 ADVANCEMENTS IN PROTEIN STRUCTURE PREDICTION USING DEEP LEARNING

**FIGURE 3:**
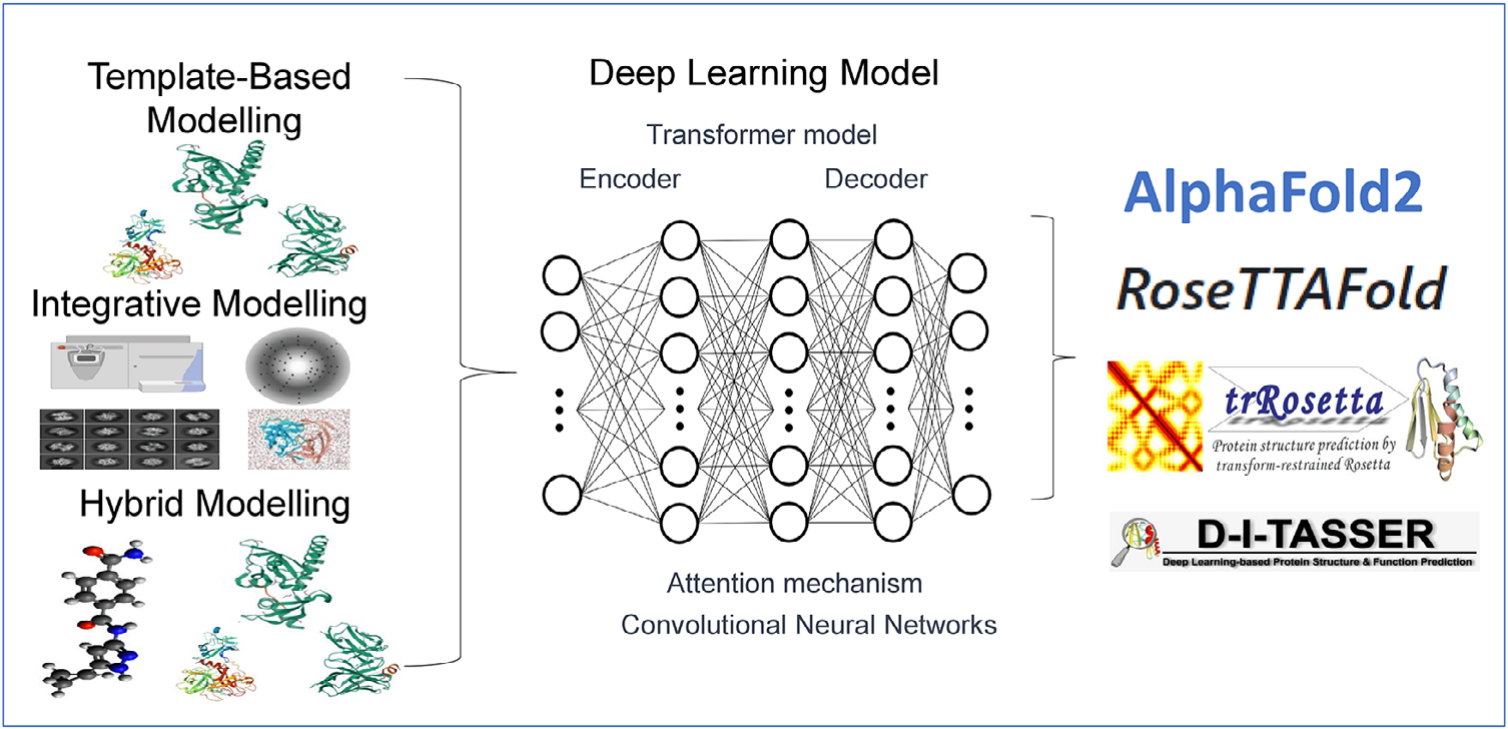
Deep learning–based protein structure prediction model.

DL‐based advancements in protein structure prediction have enabled researchers to predict the structure with greater accuracy and speed. This has important implications for drug discovery, as predicting the structure of a protein target can aid in the design of more effective drugs. The CASP provides a common platform for researchers to participate in the competition of protein structure prediction algorithm development process. It also inspired structural biologist to implement DL and transformed protein structure modelling processes with high accuracy and fast prediction. AlphaFold2 and RoseTTAFold are recent better performing DL‐based protein structure prediction tools to predict the protein structure. AlphaFold2 and RoseTTAFold leverage the strengths of fragment assembly methods, TBM and integrative approaches to achieve superior protein structure prediction accuracy. They incorporate DL to analyze sequence and structural data comprehensively, enabling them to predict protein folds even when direct homologous structures are not present in the database. General highlights of DL‐based 3D structure determination have been depicted in Figure 3.

### 4.1 AlphaFold2

**FIGURE 4:**
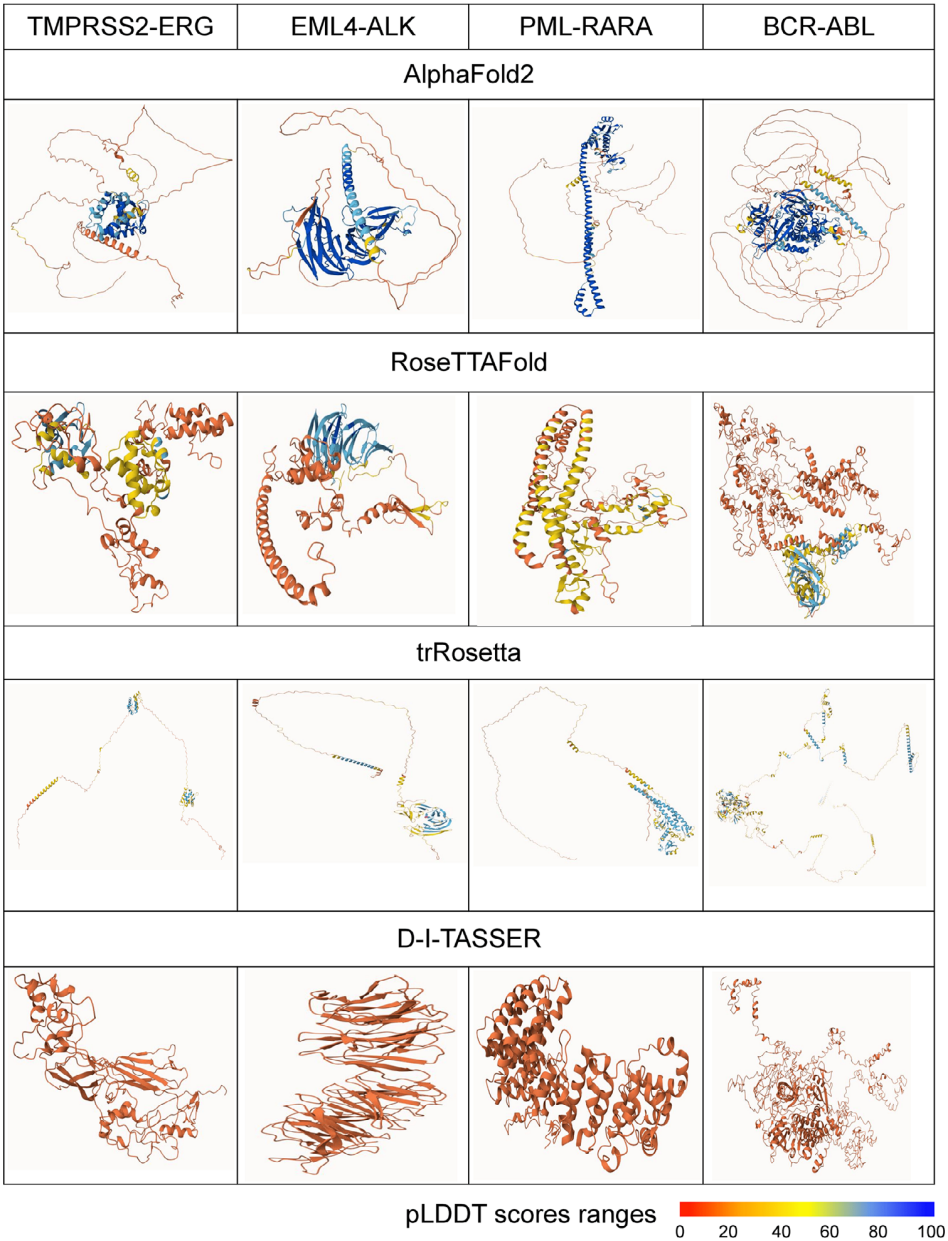
Three‐dimensional (3D) protein structure prediction and PLDDT‐based visualization of predicted structure: AlphaFold2, RoseTTAFold, trRosetta and D‐I‐TASSER with examples. TMPRSS2‐ERG, EML4‐ALK, PML‐RARA and BCR‐ABL fusion protein with corresponding PLDDT plots. Colour ranges from red to blue: red indicates lower accuracy (0–50), yellow indicates medium accuracy (51–70) and blue indicates higher accuracy (71–100).

AlphaFold2 is a DL‐based model that can predict a protein's 3D structure from its amino acid sequence.^31^, ^32^, ^72^ AlphaFold2 uses a neural network architecture that integrates evolutionary information and physical constraints to predict the structure with remarkable accuracy as shown in Figure 4.^31^ It effectively utilizes a convolutional neural network trained on protein template structures available in the PDB.^73^, ^74^ Amino acid sequences and their MSA have been used as input to the model. These inputs were used to predict the pairwise distances and torsion angles between the residues. The neural network is trained using supervised learning, where the ground truth protein structures are obtained from experimental methods like X‐ray crystallography or NMR spectroscopy. AlphaFold2 allows for simultaneous training of both stages of the network such as generation of features from the input amino acid sequence and its MSA. This is achieved by backpropagating the error signal from the final predicted structure all the way back to the input amino acid sequence, allowing the network to learn from its own errors during the structure prediction process.^72^ AlphaFold2 provides two key accuracy metrics: pLDDT and PAE scores. These metrics are used to assess the reliability of the prediction model.^32^ The pLDDT score ranges from 0 to 100, with higher scores signifying greater confidence in the accuracy of the predicted atomic positions for each amino acid residue. On the other hand, PAE scores provide the precision of inter‐residue distances, especially useful in evaluating the accuracy of the spatial relationship among different parts of the protein. Lower PAE values suggest higher confidence in the predicted distances between residues, thus indicating more accurate modelling of the protein's 3D structure.

### 4.2 RoseTTAFold

RoseTTAFold is a DL‐based model designed to predict protein structures from amino acid sequences. It uses a neural network architecture that combines residue–residue contact predictions with atomic distance predictions to achieve high accuracy.^18^ RoseTTAFold, inspired with the network architecture of AlphaFold, implemented three tracks neural network such as amino acid sequence level, distance between residues level and 3D coordinate level. Information flows back and forth among these three levels in the model to determine the relationship between a protein's chemical components and its folded structure. The network architecture involves three main components: TBM, FBM and full‐atom refinement (FAR). The TBM component of the network predicts protein structure using homologous structures as templates. It uses a transformer network to align and fuse multiple templates, and then generates an initial structure using a residue‐level graph convolutional network. The FBM component of the network predicts protein structure using fragments from the PDB that are analogous to the target sequence. It uses a transformer network to select and assemble the best fragments into a full‐length structure. Lastly, the FAR component of the network refines the predicted structure at the atomic level. It employs a recurrent neural network to optimize the geometry and energetics of the predicted structure.

### 4.3 trRosetta

trRosetta uses a neural network architecture that integrates evolutionary information and residue–residue distance predictions to achieve high accuracy.^41^ By inputting a protein's amino acid sequence into the model, a sophisticated neural network predicts detailed inter‐residue geometry, including distances and orientations. These predictions are then translated into restraints, steering structure prediction via direct energy minimization within the Rosetta framework. trRosetta is equipped to function standalone, enabling de novo prediction and facilitating extensive structure modelling.

### 4.4 D‐I‐TASSER

D‐I‐TASSER is a method used to predict protein structure with high accuracy.^75^ First, it uses deep neural networks to generate maps of inter‐residue contact, distance and hydrogen‐bond networks. One of these networks is the attention potential, which is based on the MSA transformer. Simultaneously, D‐I‐TASSER identifies structural templates using LOMETS3, a meta‐threading approach that includes models from AlphaFold2. Finally, atomic models are created through iterative fragment assembly Monte Carlo simulations, guided by the I‐TASSER force‐field and DL constraints. The biological functions of the protein are inferred from these structural models.^42^

In Figure 4, we conducted the 3D structure prediction of fusion proteins, and as an example (i) TMPRSS2‐ERG, (ii) EML4‐ALK, (iii) PML‐RARA and (iv) BCR‐ABL, through these four most popular prediction models such as AlphaFold2, RoseTTAFold, trRosetta and D‐I‐TASSER. The visualization of the protein structure shows the corresponding pLDDT scores predicted by these prediction models. In the context of selected four fusion proteins, our analysis revealed a noteworthy trend in pLDDT scores. Specifically, we observed that the pLDDT scores tend to be higher when the structure templates of the constituent proteins are readily available in the database. When the structural templates for fusion partners are not present, the pLDDT scores for the overall fusion protein structure exhibit comparatively lower values. This observation underscores the influence of template availability on the confidence and accuracy of pLDDT‐based predictions for fusion proteins as shown in Figure 4B. We also observed that out of four prediction models, AlphaFold2 performs with better accuracy as its pLDDT scores are higher (blue) for all four fusion proteins.

**FIGURE 5:**
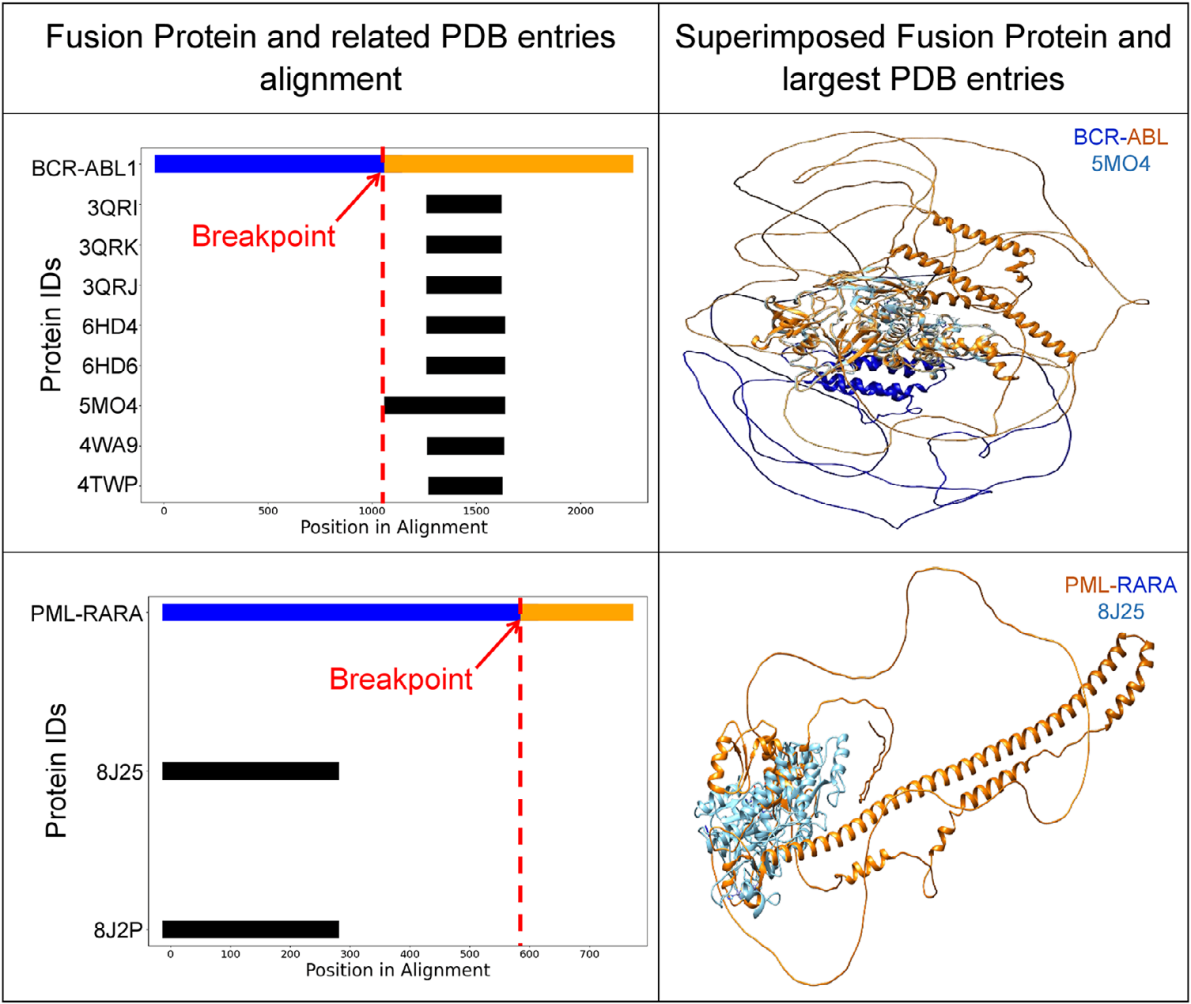
Comparison of known structures and our predicted fusion proteins in the protein sequence alignment and three‐dimensional (3D) structure superimpose. Left Panels: Alignment of the BCR‐ABL (top) and PML‐RARA (bottom) fusion proteins with their related Protein Data Bank (PDB) entries. The BCR‐ABL fusion protein (blue and orange) is aligned against PDB entries 3QRI, 3QRK, 3QRJ, 6HD4, 6HD6, 5MO4, 4WA9 and 4TWP, whereas the PML‐RARA fusion protein (blue and orange) is aligned against PDB entries 8J25 and 8J2P. The red dashed line indicates the fusion point in each protein. Right Panels: Superimposed structures of the fusion proteins with their largest corresponding PDB entries.

To systematically analyze fusion proteins, we performed a detailed search in the PDB database. We employed advanced search parameters to locate relevant PDB entries for each fusion protein. For BCR‐ABL, EML‐4‐ALK, TMPRSS2‐ERG and PML‐RARA, we found several entries by searching for exact phrases in the citation titles. After identifying the relevant PDB entries, we aligned these entries against the respective fusion proteins using a custom plotting script. This alignment visually highlights the breakpoint regions, providing a clear demarcation of the fusion points. The plots illustrate how the fusion proteins correspond to their structural counterparts in the PDB, emphasizing the structural continuity and potential functional regions of the fusion proteins as shown in Figure 5. The importance of these analyses lies in validating the predicted fusion protein structures against experimentally determined PDB structures. The breakpoints, indicated in the plots, are critical as they represent the regions where two different proteins fuse. This structural insight aids in designing targeted therapies and understanding the molecular mechanisms underlying fusion protein‐related disease.

## 5 COMPUTATIONAL EVALUATION OF FUSION PROTEIN 3D STRUCTURES

**FIGURE 6:**
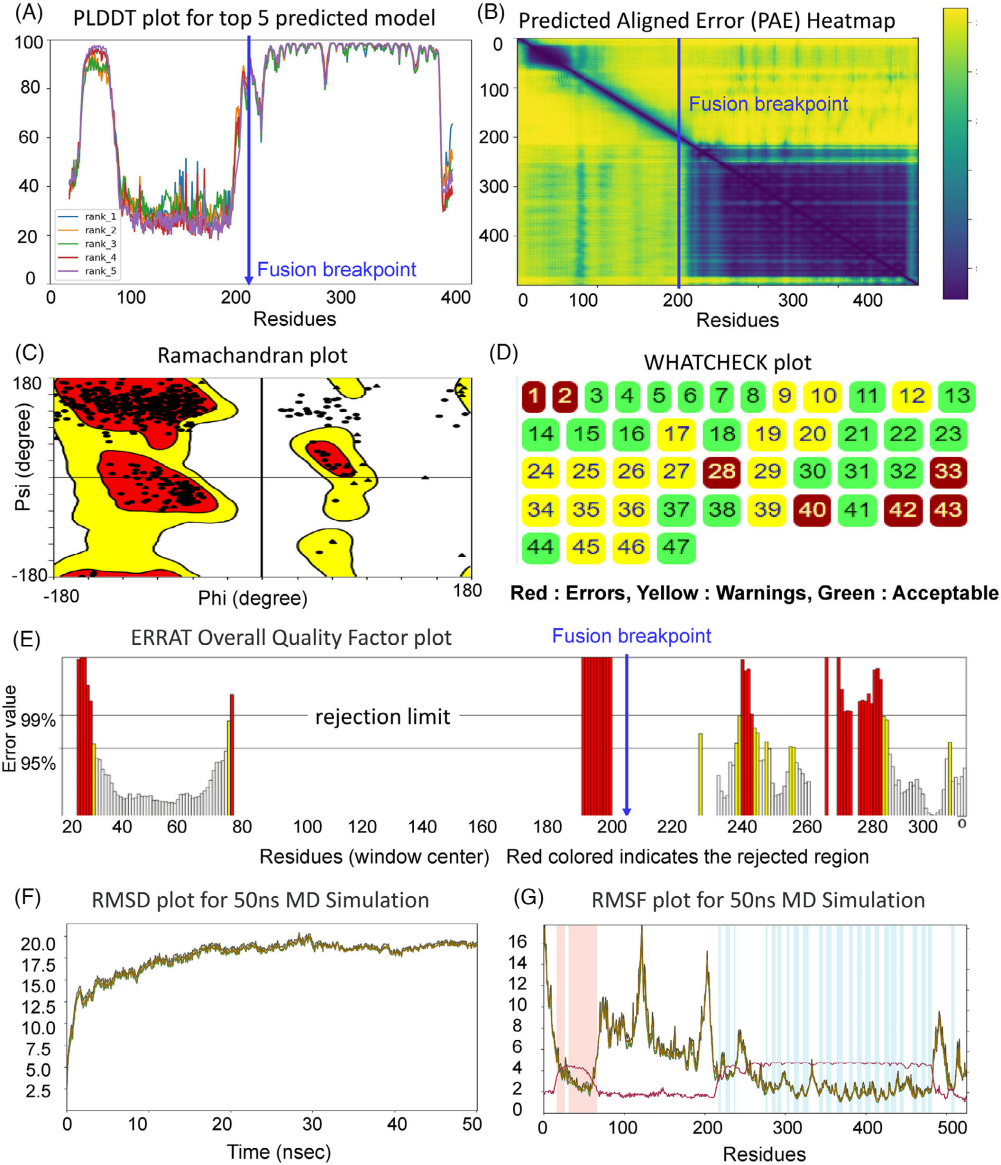
In silico assessment and validation of EML4‐ALK fusion protein. (A) PLDDT plot of top five models by AlpahFold2, (B) predicted alignment error plot (PAE), (C) Ramachandran plot, (D) WHATCHECK (each number corresponds to a specific check or validation parameter such as bond angles, bond lengths, planarity and torsion angles.), (E) ERRAT plot, (F) molecular dynamics simulation of fusion protein till 50 ns through Desmond. The left panel shows the root mean square deviation (RMSD) plot of C‐alpha, backbone, side chain and heavy atoms and (G) the right panel shows the root mean square fluctuation (RMSF) plot of C‐alpha, backbone, side chain, heavy atoms and B factor. Colour shading: red indicates alpha helices, and blue indicates beta helices.

Protein 3D structure refinement involves improving the accuracy of an experimentally or computationally predicted protein structure. This refinement process is necessary because protein structures obtained through experimental or computational methods are often imperfect and contain errors. There are several approaches to protein structure refinement, including energy minimization, MD simulations and machine learning–based methods.^76^ These methods aim to optimize the protein structure by minimizing the energy of the system or enhancing the accuracy of the predicted structure.^77^ In recent years, DL‐based methods have demonstrated promising results in the refinement of protein structures.^77^, ^78^ These methods employ neural networks to learn the underlying patterns and features of protein structures, refining the structure based on these learned patterns.^76^, ^79^ The application of DL‐based methods has significantly enhanced the accuracy of protein structure prediction and holds the potential to further advance the field of structural biology. We used some of the known assessment tools such as pLDDT, PAE, Ramachandran plot, WHATCHECK, ERRAT and MD simulations to analyze the accuracy of fusion protein structure prediction and shown in Figure 6. Table 2 summarizes the key features and applications of these refinement tools. Verify3D, ERRAT, PROCHECK, ProSA and MolProbity each provide unique methods for assessing structural compatibility, stereochemical quality and overall geometry of the protein models. These tools help to ensure that the predicted structures are consistent with known protein structures and are accurate representations of the fusion proteins. Each tool's limitations and their applicability to fusion proteins are also discussed, highlighting the importance of using multiple validation approaches to obtain reliable structural models. Similarly, Table 3 presents the validation scores for the predicted fusion protein structures using various computational tools. The table compares the performance of AlphaFold, D‐I‐TASSER, RoseTTAFold and trRosetta across four fusion proteins: BCR‐ABL, EML4‐ALK, TMPRSS2‐ERG and PML‐RARA. The validation tools ERRAT, VERIFY3D and PROCHECK were used to assess the quality of the predicted structures. Each tool provided distinct percentage scores, reflecting different structural accuracies.

**TABLE 2:** Overview of refinement tools used for validating fusion protein structures.

Refinement tools	Features	Application in fusion protein	Reference
Verify3D	Evaluates compatibility of predicted protein structure with its amino acid sequence Uses a statistical potential derived from high‐quality protein structures Scores each residue based on accommodation in its environment. Provides a graphical representation of compatibility scores for each residue	May not align perfectly with statistical potential derived from traditional protein structures due to unique structural features of fusion proteins Scoring system may not account for irregularities or non‐standard conformations in fusion proteins. Accuracy influenced by the completeness of the predicted structure and the availability of relevant reference structures	^80^
ERRAT	Compares predicted protein structure to a set of high‐resolution structures Calculates the overall quality factor based on non‐bonded interactions. Employs an empirical R‐factor measuring agreement with reference structures	Sensitivity to smaller errors and inability to account for unique characteristics of fusion proteins. It may not encompass the diversity of fusion protein structures, leading to potential validation inaccuracies	^81^
PROCHECK	Checks stereochemical quality of protein structures Evaluates geometry of residues, packing and presence of unusual features Generates Ramachandran plot for backbone torsion angles analysis	Reliance on parameters from high‐resolution structures may not fully represent fusion proteins' characteristics Primarily evaluates geometric aspects, potentially missing context‐specific features relevant to fusion proteins	^82^
ProSA	Evaluates structure quality by comparing to known protein structures Uses machine learning to assess structure based on energy profile. Calculates *Z*‐score indicating structure's fit with expected energy distribution	Unique structural arrangements of fusion proteins may deviate from traditional energy distributions, affecting *Z*‐score accuracy Limited availability of similar reference structures for less‐studied fusion protein variants	^83^
MolProbity	Checks for steric clashes and evaluates overall geometry Calculates validation parameters like clash score, rotamer outliers. Utilizes geometric and energetic criteria for structure assessment	Useful for identifying potential structural inaccuracies. Provides detailed visualization options for structure exploration and error identification	^84^

**TABLE 3:** Heatmap representation of the validation metrics of SAVES server.

Prediction tool	Fusion protein	Validation tools (%)		
		ERRAT	VERIFY3D	PROCHECK
**AlphaFold**	BCR‐ABL	88.79	50.42	63.5
	EML4‐ALK	92.83	49.62	78.6
	TMPRS‐ERG	93.42	43.87	69.1
	PML‐RARA	93.01	23.92	75.3
**D‐I‐TASSER**	BCR‐ABL	70.21	59.25	48.60
	EML4‐ALK	77.60	64.20	58.50
	TMPRS‐ERG	64.40	64.93	48.90
	PML‐RARA	90.30	50.67	74.20
**RoseTTAFold**	BCR‐ABL	11.80	47.70	45.20
	EML4‐ALK	6.80	42.20	64.30
	TMPRS‐ERG	13.80	33.30	48.20
	PML‐RARA	7.60	25.70	58.90
**trRosetta**	BCR‐ABL	86.50	48.50	90.40
	EML4‐ALK	81.00	52.00	86.50
	TMPRS‐ERG	91.10	56.30	92.30
	PML‐RARA	90.10	44.30	89.00

*Note*: The metrics evaluated are ERRAT, VERIFY3D and PROCHECK scores. Higher validation scores are depicted in darker shades of red, indicating better structural quality, whereas lighter shades represent lower scores. This visualization aids in comparing the performance of different prediction tools across the fusion proteins BCR‐ABL, EML4‐ALK, TMPRSS2‐ERG and PML‐RARA.

## 6 REFINEMENT AND ASSESSMENT OF FUSION PROTEIN STRUCTURE PREDICTION THROUGH MOLECULAR DYNAMICS (MD) SIMULATION

Protein structure refinement is a process aimed at improving the accuracy and quality of predicted or experimentally determined protein structures. The goal is to align the model more closely with the true atomic structure of the protein. There are several approaches to protein structure refinement, including energy minimization, MD simulations, knowledge‐based refinement and hybrid methods. There are several tools available for protein structure refinement. Table 4 presents a summary of the leading computational tools utilized for the refinement of protein structures, encompassing various methodologies such as homology modelling, MD and experimental data integration. Overall, protein structure prediction is crucial in MD simulations of predicted structures or protein–ligand complexes. MD simulations provide insights into the mechanisms of ligand binding, revealing key interactions between the protein and ligand, conformational changes in the protein upon binding and the role of water molecules in the binding process. Experimental techniques, like X‐ray crystallography, NMR spectroscopy and cryo‐EM, can determine protein structures and serve as benchmarks for validating predicted structures. There are several softwares available for MD simulations, both open source and commercial as shown in Table 4. To understand the predicted structures accuracy and their stability pattern into the system, we performed MD simulation for BCR‐ABL, EML4‐ALK, TMPRSS2‐ERG and PML‐RARA fusion proteins till 50 ns. We used Desmond module of Schrodinger such as system builder, MD simulation and simulation interaction panel for MD simulation. The root mean square deviation (RMSD) and root mean square fluctuation (RMSF) plots of in Figure S1, (A) TMPRSS2‐ERG, (B) EML4‐ALK, (C) PML‐RARA and (D) BCR‐ABL1. It includes RMSD plots over 50 ns time to assess protein stability and RMSF plots against residue index to evaluate amino acid flexibility, with B‐factor overlays indicating dynamic regions.

**TABLE 4:** List of tools to perform molecular dynamics simulation and refinement with their important features and related citations.

Tools	Features/strengths/limitations	References
GROMACS	A widely used open‐source simulation package for molecular dynamics and energy minimization. It is designed to simulate the Newtonian equations of motion for systems with hundreds to millions of particles. It excels in parallel performance and efficiency, employing advanced algorithms for non‐bonded interactions	^85^
AMBER	A package that provides a suite of tools for carrying out molecular simulations, including molecular dynamics and energy minimization. It also allows to study binding affinities and conformational changes in biomolecules with high precision	^86^
NAMD	A parallel molecular dynamics code that provides high‐performance simulations of large biomolecular systems. It supports scalable parallel processing, enabling simulations of complex systems on supercomputers and multi‐core workstations. It also integrates with multiple force fields and simulation tools, ensuring compatibility and flexibility in setting up and analyzing molecular dynamics simulations	^87^
CHARMM	A simulation package for molecular dynamics and other computational chemistry applications. It supports a wide range of force fields and advanced simulation techniques, making it suitable for studying complex biomolecular systems	^86^
LAMMPS	A highly scalable and parallel MD simulation code that can be used for a variety of systems and interactions. It supports a broad range of potentials and interaction types, allowing for customizable and efficient simulations of complex materials	^88^
OpenMM	An open‐source toolkit for molecular simulation that provides GPU acceleration and supports a range of molecular mechanics force fields. It supports a variety of molecular mechanics force fields, making it suitable for a wide range of biomolecular simulations	^88^
Desmond	A commercial molecular dynamics package integrated within the Schrödinger suite, widely used in drug discovery and molecular modelling. It offers high performance and scalability for simulating complex biomolecular systems with advanced algorithms and efficient parallelization	^89^
Rosetta	A software suite that employs physics‐based energy functions and machine learning for high‐accuracy structure predictions. It is widely used for protein structure prediction, protein design and docking studies	^46^
CNS	A program for structure refinement using X‐ray crystallography and NMR spectroscopy data. It integrates molecular dynamics simulations to improve the accuracy of macromolecular structure determination	^90^
PHENIX	A suite for macromolecular structure determination, including protein structure refinement modules. It provides tools for automated crystallographic and cryo‐EM structure solution and refinement	^91^

Abbreviations: Cryo‐EM, cryo‐electron microscopy; MD, molecular dynamic; NMR, nuclear magnetic resonance.

In the provided plot of PML‐RARA fusion proteins in Figure 6F, we depict the RMSD evolution of the protein, shown on the left *Y*‐axis. To generate this, all frames of the protein undergo an initial alignment based on the reference frame's backbone, followed by RMSD calculation using a specific atom selection. Monitoring RMSD offers valuable insights into the structural conformation of the protein throughout the simulation. It acts as an indicator of equilibration, showing fluctuations around a thermal average structure as the simulation progresses. For small, globular proteins, RMSD changes within the range of 1–3 Å are typically deemed acceptable. However, significantly larger changes in RMSD suggest substantial conformational alterations occurring during the simulation. Ideally, RMSD values should stabilize around a constant value, indicating that the system has reached equilibrium. If, towards the end of the simulation, the protein's RMSD is still showing consistent increases or decreases, it may indicate incomplete equilibration, and the simulation might require further duration for rigorous analysis. Out of the four example fusion proteins, PML‐RARA shows more fluctuations during the entire run of the MD simulation. Further investigation is needed to assess the specific impact of the fusion on the simulation's stability and to address potential strategies for achieving more consistent results in the case of the PML‐RARA fusion protein.

Furthermore, in Figure 6G, the RMSF analysis of PML‐RARA fusion proteins provides valuable insights into local structural dynamics along the protein chain. RMSF assesses variations in terms of C‐alpha atoms, backbone atoms, side chain atoms, heavy atoms and B‐factors, offering a comprehensive view of how individual regions of the protein exhibit flexibility and fluctuations. This analysis enables a detailed examination of local conformational changes, enhancing our understanding of protein dynamics and function. As shown in the right panel of Figure 6G, alpha‐helical regions are highlighted with a red background, whereas beta‐strand regions are highlighted with a blue background. The B‐factor plot of our predicted fusion protein reveals fluctuations in atomic mobility and thermal vibrations across the protein structure. These fluctuations are indicative of dynamic conformational changes within the fusion protein. The presence of fusion events often introduces novel structural elements and interactions, leading to varying degrees of flexibility in different regions of the protein. The observed fluctuating B‐factor plot suggests that the fusion protein may undergo dynamic structural transitions during the simulation, highlighting the importance of studying its conformational dynamics in detail to gain insights into its functional behaviour. However, the analysis of these predicted fusion protein revealed notable inconsistencies in the fluctuation profiles across all structural parameters, including C‐alpha, backbone, side chain and heavy atoms. These irregular fluctuations collectively suggest a complex and dynamic behaviour of the fusion protein during the simulation. Such fluctuations may be attributed to the intricate interplay of structural elements resulting from the fusion event, leading to unpredictable conformational changes across different regions of the protein as shown in RMSF plot. These observations underscore the need for a thorough investigation of the fusion protein's structural dynamics to gain a comprehensive understanding of its functional implications.

## 7 IMPORTANCE OF ACCURATE 3D STRUCTURES OF FUSION PROTEINS

Overall, predicting drug binding with predicted protein structures is a challenging task that requires accurate modelling of both the protein and ligand structures, a thorough understanding of protein–ligand interactions and consideration of the flexibility of both the protein and the ligand. Although there have been significant advancements in computational methods for predicting drug binding, there is still much work to be done to improve the accuracy and reliability of these predictions specially for fusion protein targets. Some of the difficulties include accuracy of the predicted protein structure, protein flexibility, water molecules, ligand flexibility and protein–ligand interactions. Understanding the nature of the interactions between the protein and the ligand is crucial for predicting binding. Accurately modelling these interactions can be challenging, especially for large and complex ligands. Fusion proteins often have complex 3D structures that can be difficult to predict, especially if the fusion partners have no previously characterized structures. This can make it hard to identify potential drug–binding sites based on structural features. Fusion proteins often have multiple domains or subunits with different functions and binding partners, which can make it difficult to identify a specific binding site for a drug. In some cases, the drug may need to target a specific interface between the fusion partners to have an effect, which can be difficult to predict or identify. Fusion proteins may have dynamic or flexible regions that can change conformation depending on their binding partners or other environmental factors. This requires the use of specialized software and hardware, as well as careful optimization of simulation parameters.

## 8 FUSION PROTEIN PRODUCTION, PURIFICATION AND EXPERIMENTAL TECHNIQUES

**FIGURE 7:**
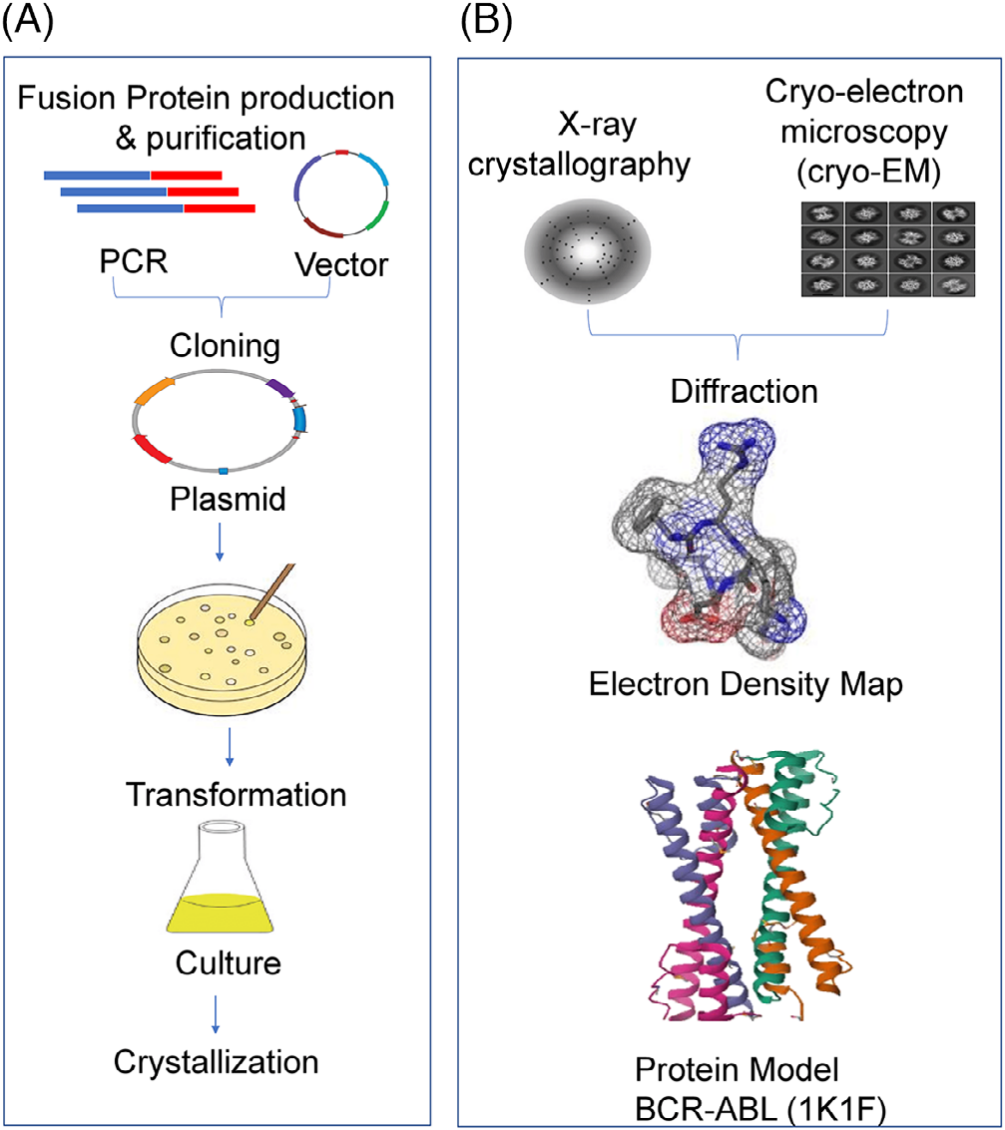
Schematic representation of experimental production, purification and validation of fusion protein structure. (A) Fusion protein production and purification (B) X‐ray crystallography and cryo‐electron microscopy for structure prediction.

The experiment of fusion proteins for the production and purification typically involves the genetic engineering of host cells (*Escherichia coli*) to express the fused genes, followed by the induction of protein synthesis.^92^ After expression, cell lysates or culture supernatants are processed to extract the fusion proteins. Various purification techniques, such as affinity chromatography or ion exchange chromatography,^92^ are employed to isolate the target fusion proteins from other cellular components. These purified proteins can then undergo for functional assays or structural studies, as shown in Figure 7A. Following purification, the obtained fusion proteins undergo for structural elucidation using techniques like X‐ray crystallography or cryo‐EM. In X‐ray crystallography, purified protein crystals are exposed to X‐ray beams, producing diffraction patterns that can be analysed to determine the 3D atomic structure of the protein, as shown in Figure 7B. Achieving high‐quality, homogeneous protein samples in sufficient quantities for crystallography can be difficult, as the process requires the formation of good crystals that diffract X‐rays effectively. This step might be frequently hindered by the chance of fusion proteins to misfold or aggregate. Cryo‐EM, while not requiring crystallization, still demands highly pure and stable protein samples for accurate structural determination, and the technique's resolution can be limited by the size and conformational flexibility of the fusion protein. Additionally, the computational reconstruction of cryo‐EM images into a coherent structure involves high end data processing and interpretation, often challenged by the complex and dynamic nature of fusion proteins. These technical and methodological challenges hinder innovation in both experimental and computational approaches, limiting our ability to fully understand the structure and function of fusion proteins.

## 9 PERSPECTIVES

Fusion protein structure prediction can be challenging due to several factors, including the complex nature of the fusion protein, the diverse range of possible conformations and the limited availability of experimental data. Additionally, fusion proteins often contain domains with distinct structural characteristics, complicating the prediction process. Several approaches have been developed to address these challenges. Techniques, like X‐ray crystallography, NMR spectroscopy and cryo‐EM, are frequently employed. However, these methods can be time‐consuming, costly and often constrained by the protein's size and complexity. Computational methods have also been developed to predict the structure of fusion proteins. In this review paper, we utilized advanced computational approaches to predict the structures of fusion proteins BCR‐ABL, EML4‐ALK, TMPRSS2‐ERG and PML‐RARA, aiming to explore the challenges faced by advanced prediction models. When examining fusion proteins with fusion partner structures absent from the database, we noted a decrease in PLDDT scores for the corresponding regions in most of the tools. This decrease in scores suggests reduced accuracy in the prediction model's performance when applied to fusion proteins with unique structural components. We predicted the 3D structures of well‐known fusion proteins using AlphaFold2, RoseTTAFold, tr‐Rosetta and D‐I‐TASSER. From this study, we identified that some protein functional domains were consistently well predicted by all four AI tools. This might be for these domains having well‐conserved 3D structures or being present in the extensive training datasets of protein structure databases, enabling the models to learn their conformations accurately. This is significantly impactful for the drug discovery process. Accurately predicted domains can serve as reliable targets for drug‐binding studies, facilitating the identification of potential therapeutic compounds. The consensus of these different tools may be helpful to enhance confidence of the 3D structure prediction.

**FIGURE 8:**
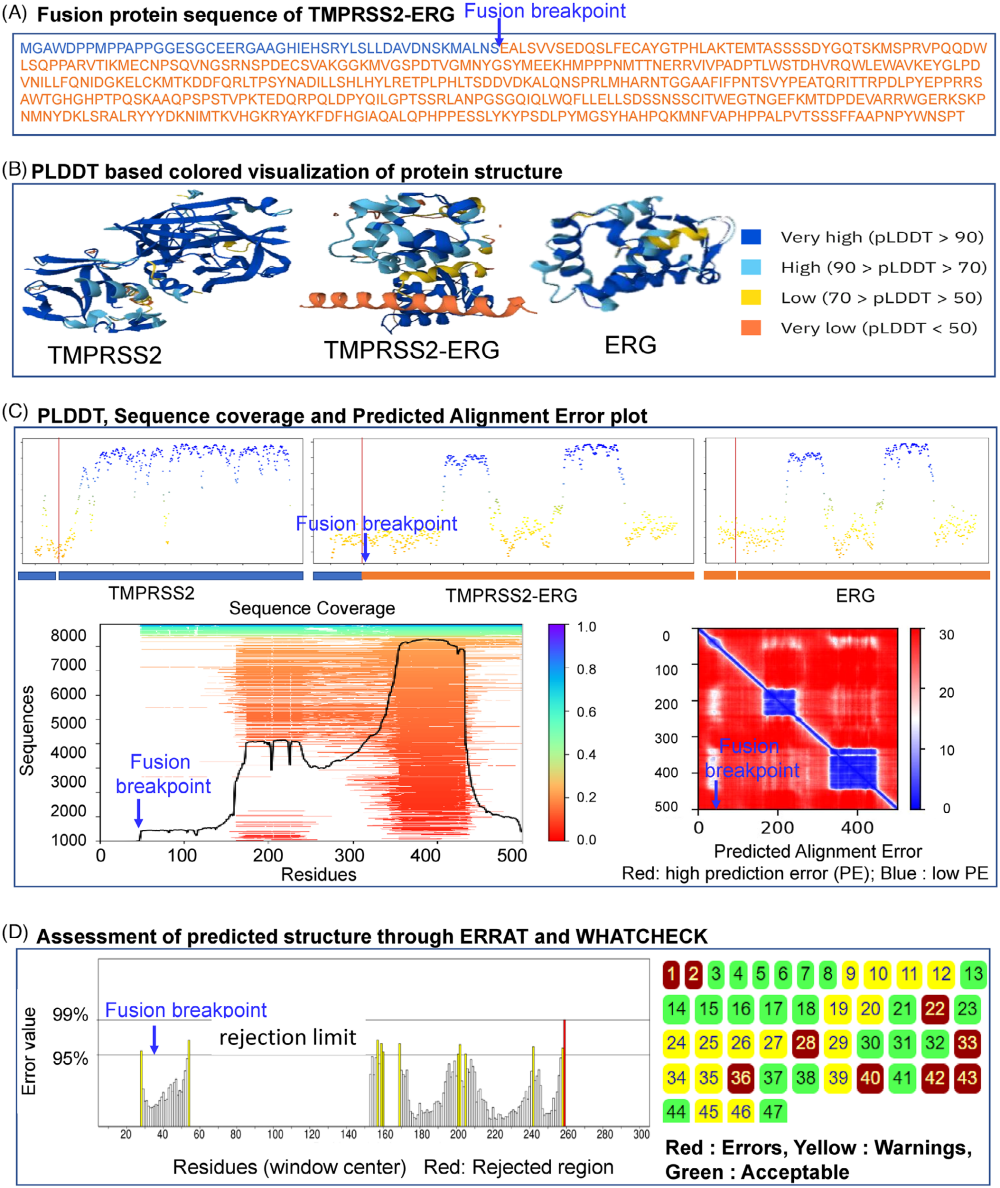
An example of sequence‐based fusion protein structure prediction, accuracy and validation of TMPRSS2‐ERG. (A) Sequence of fusion protein TMPRSS2‐ERG, (B) three‐dimensional (3D) structures by AlphaFold2, (C) confidence, coverage and PAE of the prediction model and (D) prediction of model assessment, ERRAT: The ERRAT plot shows the quality of the protein model by evaluating the non‐bonded atomic interactions. The *Y*‐axis represents the error values, and the *X*‐axis represents the residue position. Yellow bars: Residues with error values between 95% and 99%, indicating moderately reliable regions. Red bars: Residues with error values above 99%, indicating potentially unreliable regions. Grey bars: Residues with error values below 95%, indicating highly reliable regions. WHATCHECK: The WHATCHECK plot evaluates the quality of the protein structure by analyzing various geometrical parameters. Each square represents a different residue or region within the protein. Green squares: Regions with no errors, indicating a well‐modelled structure. Yellow squares: Regions with minor errors, suggesting potential areas for improvement. Red squares: Regions with significant errors, indicating potentially unreliable regions in the structure. The numerical values correspond to various geometric parameters, with colour coding reflecting the reliability of each region based on the WHATCHECK analysis.

During MD simulation analysis result, higher fluctuation in some of the fusion proteins during 50 ns MD simulation also indicates the limitations of accuracy of DL model. As an example, we have shown details of TMPRSS2‐ERG fusion protein in Figure 8. This figure presents a comprehensive analysis of the TMPRSS2‐ERG fusion protein. Panel A depicts the amino acid sequence of the fusion, with the breakpoint highlighted. Panel B showcases the protein structure, color‐coded by the pLDDT scores, indicating regions of high to very low confidence. Panel C provides a fusion breakpoint analysis through sequence coverage and predicted alignment error and pLDDT, and Panel D evaluates the predicted structure's accuracy using the ERRAT and WHATCHECK validation tools, highlighting regions of error and warning, and areas deemed acceptable. In our approach, it is important to acknowledge certain limitations, particularly in the context of DL and AI predictions. One significant concern is that if our datasets predominantly contain representations of WT folded proteins, the models might confidently predict structures resembling the WT proteins rather than accurately reflecting the true structure of the fusion proteins. This potential bias arises because the fusion proteins represent a distinct encoded product, which may not be well represented in the training data. Consequently, although DL/AI tools offer powerful predictive capabilities, there is a risk that the predictions may not fully capture the unique structural features of fusion proteins. This limitation underscores the need for careful validation and possibly experimental verification to ensure the reliability of the predicted fusion protein structures. To improve the accuracy of fusion protein structure prediction, it is important to use a combination of experimental and computational approaches. Additionally, incorporating data from multiple sources, such as sequence homology, structural data and functional information, can also improve the accuracy of predictions. The 3D structure prediction research directions through AI include integrating multi‐modal data (such as genomic, proteomic and transcriptomic information) to enhance predictive accuracy. Developing hybrid models that combine AI with experimental data can also improve reliability. Advancements in AI models to predict the effects of point mutations on protein structure are also one of the crucial points. The advancements towards the explainable AI approaches in this study field may provide deeper insights into the underlying mechanisms of protein folding. Of course, increasing the size and diversity of training datasets is also important for refining AI models and overcoming current prediction challenges. We hope we can have more accurate AI model in the protein 3D structure prediction not only for the WT/single protein but also for the new proteins made by DNA double‐strand breakage (Figure 7).

## AUTHOR CONTRIBUTIONS

**Himansu Kumar and Pora Kim**: Design; writing; figures; review and editing.

## CONFLICT OF INTEREST STATEMENT

The authors declare no conflicts of interest.

## ETHICS STATEMENT

Not applicable.

## Supporting information


**Figure S1 Molecular dynamics simulation plots for four fusion proteins**. (A) TMPRSS2‐ERG, (B) EML4‐ALK, (C) PML‐RARA and (D) BCR‐ABL1. Left Panels: root mean square deviation (RMSD) plots over a 50 ns simulation period, showing the stability of the protein structures. Right Panels: root mean square fluctuation (RMSF) plots against residue index, illustrating the flexibility of amino acids. Overlays of B‐factor values highlight dynamically flexible regions within the proteins. Colour shading in RMSF plots: Red indicates alpha helices, and blue indicates beta sheets.

**Table S1. Catalogues the in‐frame fusion genes identified in the study, listing the 5′ and 3′ gene partners and the corresponding number of articles reported in PubMed**.

## Data Availability

Data are available on reasonable request from the corresponding author.
