# Supplementary figures and images for: Artificial intelligence in fusion protein three‐dimensional structure prediction: Review and perspective
Source: Clin Transl Med. 2024 Aug 1;14(8):e1789. doi: 10.1002/ctm2.1789 (PMC11294035; doi:10.1002/ctm2.1789)

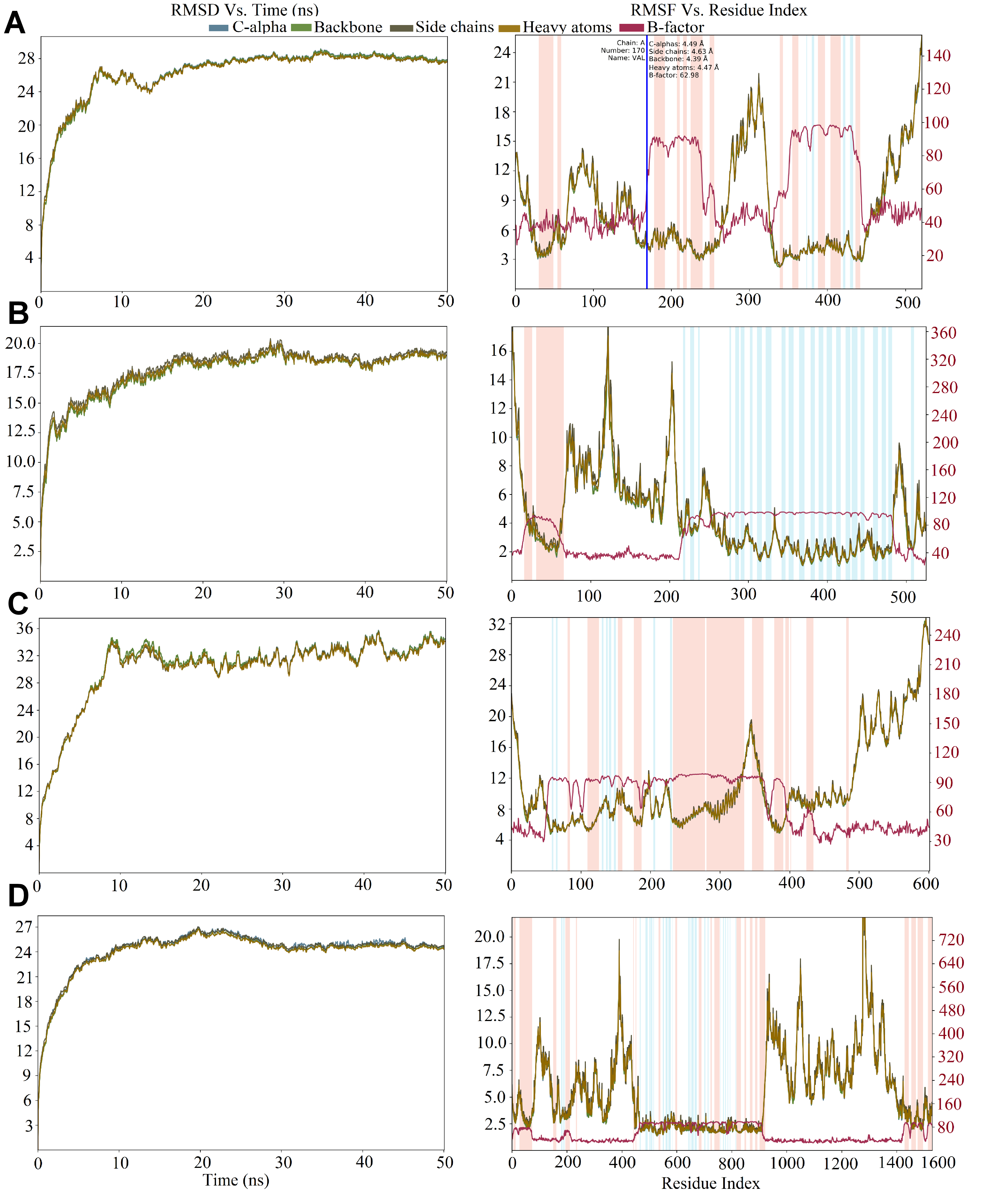

Supplement: Supplementary file 1 — Figure S1 Molecular dynamics simulation plots for four fusion proteins. (A) TMPRSS2‐ERG, (B) EML4‐ALK, (C) PML‐RARA and (D) BCR‐ABL1. Left Panels: root mean square deviation (RMSD) plots over a 50 ns simulation period, showing the stability of the protein structures. Right Panels: root mean square fluctuation (RMSF) plots against residue index, illustrating the flexibility of amino acids. Overlays of B‐factor values highlight dynamically flexible regions within the proteins. Colour shading in RMSF plots: Red indicates alpha helices, and blue indicates beta sheets. [file CTM2-14-e1789-s001.png]
